# Tofacitinib to Treat Severe Acute Refractory Colitis in a Teenager: Case Report and Review of the Literature

**DOI:** 10.1097/PG9.0000000000000241

**Published:** 2022-08-16

**Authors:** Chloé Girard, Martha Dirks, Colette Deslandres

**Affiliations:** From the * Faculty of Medicine, Université de Montréal, Montréal, Québec, Canada; † Division of Gastroenterology, Department of Pediatrics, CHU Sainte-Justine, Montréal, Québec, Canada; ‡ CHU Sainte-Justine Research Center, Montréal, Québec, Canada.

**Keywords:** acute severe colitis, janus kinase inhibitor, pediatrics

## Abstract

Acute severe colitis (ASC) may occur within 3 months of ulcerative colitis diagnosis in 9%–15% of children and the rate of colectomy is up to 40%–50% within 5 years after an ASC. The aim of this publication is to present recent and relevant data on the success of medical treatment with tofacitinib in ASC.

**Methods:**

We report a challenging case of a teenage boy with ASC at diagnosis and conduct a discussion after a review of the literature regarding the use of tofacitinib in inflammatory bowel disease, especially in pediatric patients and in ASC.

**Results:**

The patient was hospitalized for 10 weeks and was refractory to conventional therapies: intravenous corticosteroids, infliximab, methotrexate, and vedolizumab. He received 7 blood transfusions and also presented with a severe malnutrition requiring a total parenteral nutrition. Tofacitinib was considered as a medical last resort before colectomy and was started at week 8. Thirteen days after starting tofacitinib, he was asymptomatic and was discharged on tofacitinib as sole treatment. By week 9 of tofacitinib, a colonoscopy showed both endoscopic and histological remission. He has remained in clinical remission at 6-month follow-up.

**Conclusions:**

Tofacitinib may be an alternative medical treatment to avoid colectomy in ASC. It is a small molecule with a rapid onset and few severe adverse events. It has been used for ASC in adult patients, allowing to avoid colectomy in more than 60%. To our knowledge, this is one of the few pediatric patients with refractory ASC at initial diagnosis who responded to tofacitinib.

**What Is known?**

- Acute severe colitis is associated with a high risk of colectomy.
- Tofacitinib is approved for the treatment of severe ulcerative colitis in adult patients.
- There are few studies in adults on the use of tofacitinib in acute severe colitis but none in pediatrics.

**What Is New?**

- Tofacitinib may be an alternative medical treatment averting colectomy in acute severe colitis in children.

## INTRODUCTION

Acute severe colitis (ASC) in children is defined by a pediatric ulcerative colitis activity index (PUCAI) ≥65 points. Nine percent to 15% of children develop ASC within 3 months of ulcerative colitis (UC) diagnosis, and during the course of disease in childhood, the incidence may be as high as 28% (1–4). Before the advent of infliximab (IFX) and calcineurin inhibitors, 40%–70% of cases of ASC underwent colectomy during hospitalization, but now the rate has decreased to 10%–20% of children. However, the 5-year colectomy rate remains at 40%–50% (5–9).

Over the past 2 years, a few studies in adults on the use of tofacitinib in ASC have been published with encouraging results, but to date, there is no data for pediatric ASC. We report a teenage patient with refractory ASC successfully treated by tofacitinib. In the discussion, we will conduct a review of the published literature on the use of tofacitinib in pediatrics and in cases of ASC in adults.

## PATIENT

A 14.5-year-old boy presented to our pediatric gastroenterology clinic with a 5-week history of bloody diarrhea with a frequency of 15 stools per day, nocturnal stools, and abdominal pain. He had not attended school for the past 3 weeks. His past medical history was negative. There was no family history of inflammatory bowel disease (IBD). He had lost 2.3 kg in 1 month, and his body mass index (BMI) at presentation was 14.9 kg/m^2^. His physical examination was normal aside from pallor. At time of presentation, C-reactive protein (CRP) was increased to 19.2 mg/L (normal <1.0 mg/L), serum albumin was 34 g/L (normal 37–47 g/L), hemoglobin was 117 g/L (normal 130–160 g/L), and white blood cell differential showed hypereosinophilia (1300/mm^3^, normal <400). Fecal calprotectin was >2100 μg/g. Stools cultures and *Clostridium difficile* toxin test were negative. Upper endoscopy showed a severe gastritis and a duodenitis with some small aphthous ulcerations; colonoscopy revealed a Mayo 3 colitis from the rectum to the transverse colon, the transition zone was very clear with a totally normal right colon and terminal ileum. Moderate chronic inflammation and numerous eosinophils were found on the gastric and duodenal biopsies, which is well described as UC-related upper gastrointestinal tract manifestation (10). Severe chronic active inflammation with no granuloma were found on the colonic and rectal biopsies. Magnetic resonance enterography revealed inflammation from the rectum to the transverse colon, without inflammation of the small bowel. The colon wall had a maximum of 4 to 5 millimeters of thickness, worst in the transverse colon.

He was admitted to the gastroenterology ward with a PUCAI of 55. After 3 days of intravenous corticosteroids (IVCS) and a first dose of IFX (5 mg/kg), he was discharged on 40 mg of prednisone die. Eight days later, he was readmitted to our hospital with a PUCAI of 70. He failed IVCS (up to 2 mg/kg/d for the 3 first weeks and tapering off at week 9), IFX (6 infusions: the first at week 0 with a dose of 5 mg/kg and the following at weeks 1, 2, 3, 6, and 7 with a dose of 10 mg/kg), subcutaneous methotrexate (2 doses of 25 mg/dose at weeks 2 and 3), vedolizumab (weeks 3, 5, and 9), 5-aminosalicylic acid (per os during week 1, intrarectal during weeks 3–5), and vancomycin per os (weeks 3 and 4). He also needed intravenous iron supplementation (weeks 0, 1, 2, 3, and 9), total parenteral nutrition (TPN) (weeks 2–9) with frequent albumin infusions in the TPN. He presented with a severe malnutrition (STRONGkids risk 5/5) (11): upon admission, his weight was 40.5 kg but he lost weight, although he was on TPN and receiving 3000 kcal intravenously. His lowest weight was 35.8 kg and BMI declined to 13 kg/m^2^ (<1st percentile/height for age). As his serum alkaline phosphatase decreased as low as 41 IU/L (normal 127–517 IU/L), he received zinc up to 14 000 mcg by day in his TPN. He required 7 blood transfusions (during weeks 3, 5, 6, 7, and 8). IFX blood concentrations remained in therapeutic range: 12.7 μg/mL before dose 2, >45 μg/mL before dose 3, 17.1 μg/mL before dose 4, and 17.7 μg/mL before dose 6, without antibody to IFX. PUCAI remained high peaking at 75 at week 4, decreasing slightly to 35 at week 6 but with persistent bloody stools and frequent nocturnal stools. A colonoscopy at week 3 showed no endoscopic improvement compared with initial colonoscopy, with a severe Mayo 3 colitis from the rectum to 50 cm from the anus. Cytomegalovirus was negative on biopsies (reverse transcription-polymerase chain reaction (RT-PCR) and immunohistochemistry).

As he failed conventional therapies and dual biologics, tofacitinib was considered as a last-resort treatment before colectomy. Unfortunately, at week 6 his parents both had a symptomatic coronavirus disease 2019 (COVID-19) infection. They were in direct contact with their son every day; his mother was in contact with him until the day before she tested positive. Our patient tested negative 3 times for severe acute respiratory syndrome coronavirus 2 by nasopharyngeal RT-PCR and never developed any symptoms of COVID-19.

At week 7, CRP was 5.4 mg/L, serum albumin was 25 g/L, and hemoglobin was 75 g/L. At week 8 (day 57 after the diagnosis), tofacitinib 10 mg per os twice daily was started. The patient improved rapidly: he stopped having nocturnal stools on day 5, he passed his first normal stool, at a frequency of 1 per day, without bleeding on day 10. On day 13, TPN was discontinued, he was in clinical remission, PUCAI was 0, and he was discharged from the hospital 70 days after his admission. Upon his discharge, CRP was 0.2 g/L, serum albumin 33 g/L, hemoglobin 86 g/L, and fecal calprotectin 531 μg/g. IFX had been discontinued at week 7, vedolizumab and corticosteroids at week 9.

He was followed in the clinic every week during the first month. He did not have any gastrointestinal symptoms. Nine weeks after beginning tofacitinib, the patient underwent a colonoscopy, which showed complete endoscopic and histological remission. CRP was normal <0.2 mg/L, serum albumin was normal 43 g/L, hemoglobin was 127 g/L, and alkaline phosphatase had normalized to 263 IU/L. Serum IFX was undetectable 2 months after discontinuing IFX. Tofacitinib was decreased to 5 mg per os twice daily after this colonoscopy.

The only side effect on tofacitinib was a mild transient hypercholesterolemia reaching a maximum of 6.1 mmol/L (N <5.0 mmol/L). We also observed a transient leucopenia with a minimum of 3.43 white blood cells/μL with 1.3 neutrophils/μL.

**FIGURE 1.**
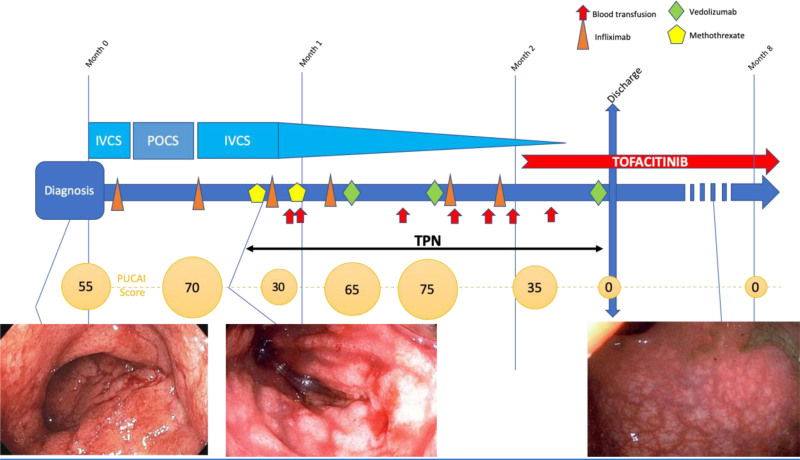
Acute severe colitis in a 14-y-old adolescent patient newly diagnosed with ulcerative colitis. IVCS = intravenous corticosteroids; POCS = per os corticosteroids; PUCAI = pediatric ulcerative colitis activity index, TPN = total parenteral nutrition.

**FIGURE 2.**
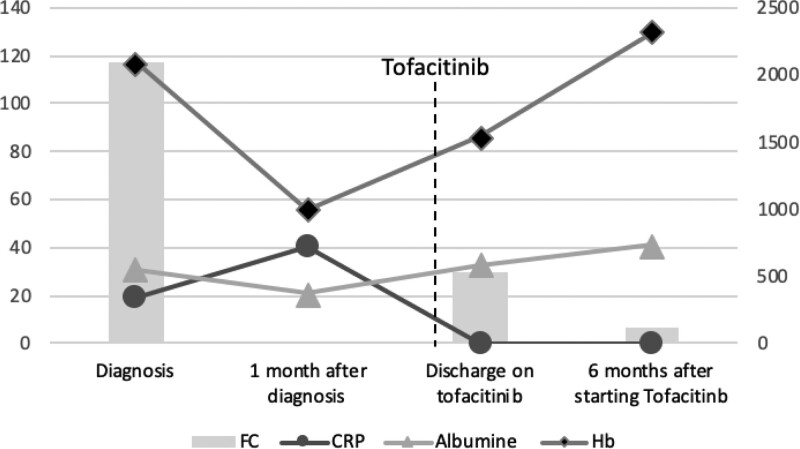
Biochemical test evolution from the diagnosis to the end of the follow-up. CRP in mg/L, Hb and serum albumin in g/L, ordinate axis on the left side; FC in μg/g, ordinate axis on the right side. CRP = C-reactive protein; FC = fecal calprotectin; Hb = hemoglobin.

At his last visit, 6 months after starting tofacitinib, he remains asymptomatic with a weight of 45.6 kg and a BMI of 16.2 kg/m^2^ (3rd percentile). Fecal calprotectin is normal: 55 μg/g, albumin is 46 g/L, CRP is <1 mg/L, and hemoglobin is 140 g/L (Figs. 1 and 2).

## DISCUSSION

The last European Crohn’s and Colitis Organization guidelines recommend that children with ASC should be admitted to hospital for intensive medical treatment with IVCS but up to one third of patients are steroid refractory (5,12). In case of nonresponse to IVCS after 3–5 days, a second-line therapy should be scheduled, and if PUCAI remains >65 on the 5th day of IVCS, a second-line therapy should be started. IFX is recommended as the second-line medical therapy for anti-tumor necrosing factor (anti-TNF) naive children, but due to rapid clearance of IFX in ASC, intensification of induction regimen and doses of IFX up to 10 mg/kg per dose are often needed to provide drug exposure equivalent to that attained with standard dosing outside the ASC setting (13–16). As a rescue therapy, the guidelines also suggested cyclosporine or tacrolimus as a bridge to long-term maintenance therapy. In general, prompt referral for urgent colectomy is recommended following failure of one second-line medical therapy (5,12,17,18). As our patient presented with ASC refractory to conventional therapy and according to the guidelines, a colectomy was discussed with the surgical team.

As the patient was inevitably heading towards a colectomy, after lengthy discussions with the parents and family about the high level of immunosuppression in our patient, we elected to proceed with a trial of tofacitinib as a last medical resort. At the start of tofacitinib, his treatment consisted of high-dose corticosteroids, IFX (last dose 4 d prior), and vedolizumab (16 d prior). Tofacitinib is a small molecule that inhibits the Janus kinases (JAKs) 1, 2, and 3 and is a central molecule in pro-inflammatory cytokine productions and response. Tofacitinib is orally administrated and non-immunogenic. Blocking the JAK signaling pathway has proven successful in other immune-mediated disorders and several oral JAK inhibitors have now received regulatory approval for the treatment of rheumatoid arthritis, psoriasis and UC in adults (19). The induction dose is 10 mg twice daily for at least 8 weeks and may be reduced to 5 mg twice daily for maintenance therapy (19–22). Data about tofacitinib in IBD are still limited and mainly in adult patients. In the Oral Clinical Trials for tofAcitinib in ulceratiVE colitis (OCTAVE) trials, tofacitinib induced remission more often than placebo in patients with moderate-to-severe UC who failed initial conventional therapy or anti-TNF therapy. Clinical remission at 52 weeks occurred in 34.3% of the patients in the 5 mg tofacitinib group and 40.6% in the 10 mg tofacitinib group versus 11.1% in the placebo group (*P* < 0.001) (23). Moreover, post hoc analysis of the trials showed that there is rapid induction of remission with these JAK inhibitors, often over a 3-day period (24). There are only a few small case series reporting the use of tofacitinib in ASC: in a total of 15 steroid-refractory adult patients, colectomy was avoided in 9 of them (27) (Table 1). In 2021, the GETAID-TALC Study Group published a multicentric study of 55 adult patients with severe refractory UC (median follow-up of 6.5 mo) treated by tofacitinib as a rescue therapy. At week 14, 33% of patients were in steroid-free clinical remission, and at 6 months, the rate of colectomy-free survival was 73.6% (95 confidence interval [CI], 61.9%–87.3%) (28). Berinstein et al (29) published a study of 40 adult patients with ASC treated with tofacitinib and concomitant IVCS. This induction strategy was effective: tofacitinib was protective against colectomy at 90 days compared with matched controls (hazard ratio, 0.28; 95 CI, 0.10–0.81; *P* = .018) and rate of complications and steroid dependence were similar between the 2 groups.

**TABLE 1.** Review of case series of adult patients with ASC treated with tofacitinib

Publication, number and characteristics of patients	Age	Patients	Duration of tofacitinib	Clinical remission	Colectomy	Response	Therapy at the end of follow-up
Berinstein et al (27) 2019	Adults	ASC1	NA	Yes	No		TOFA
Four adult patients with anti-TNF and steroid-refractory ASC		ASC2	NA	Yes	Yes		Elective colectomy for multifocal dysplasia 6 mo after tofacitinib induction
High-intensity tofacitinib: 10 mg 3 times daily and after 5 d maintenance with 5 mg 2 times daily		ASC3	NA	No	Yes	Initial rapid improvement in symptoms, but return of severe symptoms after reducing dose. Urgent colectomy necessary	Colectomy
		ASC4	NA	Yes	No	Patient developed a nonspecific truncal maculopapular rash and TOFA discontinued	VDZ
Honap et al (25) 2020	24 y	ASC1	26 wk	Yes	Yes	Steroid-free clinical remission at week 16	Colectomy at week 26 for UC exacerbation
Seven adult patients with anti-TNF and steroid-refractory ASC	55 y	ASC2	52 wk	Yes	No	Steroid-free clinical and endoscopic remission at week 53	TOFA (5 mg bid)
	33 y	ASC3	12 wk	Yes	Yes	Short clinical remission	Colectomy at week 12
	57 y	ASC4	16 wk	Yes	No	Steroid-free clinical remission	TOFA (10 mg bid)
	30 y	ASC5	2 wk	No	Yes	Nonresponse. Colectomy at week 2	
	19 y	ASC6	16 wk	Yes	No	Steroid-free clinical remission	TOFA (10 mg bid)
	42 y	ASC7	2 wk	No	Yes	Nonresponse. Colectomy at week 2	
Kotwani et al (26) 2020	25 y	ACS1	14 mo	No	No	Clinical response but no remission	TOFA/UST
	46 y	ASC2	11 mo	Yes	No	Steroid-free clinical and endoscopic remission	TOFA
Four adult patients with anti-TNF and steroid-refractory ASC	32 y	ASC3	6 mo	Yes	No	Steroid-free clinical and endoscopic remission	TOFA
	23 y	ASC4	5 mo	No	Yes	Persistent symptomatic disease and dysplasia on colon biopsies	Colectomy
Total		N = 15		Clinical remission 10/15 (67%)	Colectomy 7/15 (47%)	Steroid-free clinical remission 7/15 (47%)	

ASC = acute severe colitis; NA = not available; TNF = tumor necrosing factor; TOFA = tofacitinib; UC = ulcerative colitis; UST = ustekinumab; VDZ = vedolizumab.

Due to the short half-life (3–5 hours) of tofacitinib, adverse events related to immunosuppression and biochemical abnormalities are potentially rapidly reversible. According to the final analysis of OCTAVE trial with up to 7.0 years of treatment, the principal risks are infection and malignancy due to immunosuppression. Less frequent adverse events were pulmonary embolism, major cardiovascular events and deep vein thrombosis (30). These latter complications are the subject of a boxed warning from the US Food and Drug Administration in July 2021. These risks in pediatric patients remain unknown due to a paucity of data. Our patient did not have a family history of thrombotic disease, and he was not tested for a prothrombotic disorder. In the case of a child with a personal or family history of thrombotic disease, additional caution should be taken. In a systematic review, Ma et al (20) found 7 studies that evaluated infectious adverse effects and results were that treatment with a JAK inhibitor was associated with a significantly increased risk of infections (relative risk, 1.40; 95% CI, 1.18–1.67; *P* < 0.001). The most commonly reported infections were upper respiratory tract infections or nasopharyngitis. In the OCTAVE trials (2019), herpes zoster infections were reported to be 4 times more frequent in UC patients receiving a JAK inhibitor compare to patients receiving placebo and they reported an incidence rate of serious infections of 2 events/100 patient-years (95% CI, 1.4–2.8) (31). Additionally, previous anti-TNF exposure was not associated with an increased risk of infections. The reported risk of malignancy is 0.3% out of 2606 IBD patients who received a JAK inhibitor across all trials (mainly nonmelanomatous skin cancers). In case of dual biologic therapy in combination or with tofacitinib, a meta-analysis demonstrated overall rates of adverse events, infections and malignancy similar to historical rates of anti-TNF monotherapy (32). Elevated serum lipid concentrations were mainly reported in induction studies: compared with baseline, the mean percent change in total cholesterol ranged from 8.4% to 19.2%, with a maximum level within 6 weeks but in maintenance studies, the effect seemed to be less important with a mean percent change in total cholesterol ranged from 0.2% to 4.3%. In cases of persistent elevation, statin therapy may be started (20).

The first pediatric publication on the use of tofacitinib in IBD was in 2019. Dolinger et al (33) reported 12 children (5 Crohn disease [CD], 5 UC, 2 IBD-unclassified [IBD-U]) who received tofacitinib 10 mg twice daily for biologic-refractory IBD. Clinical response was observed in 8 patients: 5 were in clinical remission and 3 were in steroid-free clinical remission at the last follow-up after a median duration of therapy of 15.7 weeks (5–58.8 wk). There were no serious adverse effects: serum lipids were obtained in 5 patients and were normal for all. Among the 3 patients in steroid-free clinical remission, 2 had a combination therapy with tofacitinib and vedolizumab and 1 was only on tofacitinib. The same group published on dual therapy with 2 biologic agents or 1 biologic agent and a small molecule: 16 children (9 UC/IBD-U, 7 CD) were treated with vedolizumab/tofacitinib (56%), ustekinumab/vedolizumab (25%), and ustekinumab/tofacitinib (19%) after failing ≥ 2 biologic therapies, but none had ASC. Twelve patients (7 UC/IBD-U, 5 CD) achieved a steroid-free remission at 6 months including 9 patients (6 UC/IBD-U, 3 CD) on dual therapy with tofacitinib and vedolizumab (n = 7) or ustekinumab (n = 2). Only 1 patient had a severe adverse event: septic arthritis and deep vein thrombosis on vedolizumab/tofacitinib and 30 mg prednisone daily, but after specific treatment, he achieved complete mucosal healing and steroid-free remission (34). More recently, Moore et al (35) published a retrospective study including 21 patients aged 21 years and younger who started tofacitinib for medically refractory IBD (18 UC/IBD-U, 3 CD). At the end of the 12-week induction period, 9 of 21(42.9%) subjects showed clinical response, 7 of 21(33.3%) were in steroid-free remission, and 4 of 21 underwent colectomy before completing induction. After week 24, 2 other patients underwent colectomy, and 1 subject developed a sterile intra-abdominal abscess. Of the 10 patients remaining on tofacitinib at 52 weeks, 7 of 21(33.3%) were in steroid-free clinical remission. There were no instances of thrombi, zoster reactivation, or clinically significant hyperlipidemia (Table 2). We also found 2 case reports of teenagers successfully treated with tofacitinib without adverse effect: a 13-year-old girl with severe UC who had secondary loss of response to biologics who was successfully treated with tofacitinib 5 mg twice daily and went into clinical, endoscopic, and steroid-free remission after 9 months of tofacitinib and a 16-year-old female with ileocolonic CD and severe pancolitis who showed histologic remission and resolution of suspected dysplasia on 5 months of tofacitinib (36,37). The only minor adverse event in our patient was transient mild hypercholesterolemia, which had completely resolved at his last follow-up, 6 months after initiating tofacitinib. We realized that when tofacitinib was begun, he had multiple biologics on board, but we are convinced the tofacitinib was the game changer. His clinical status changed or deteriorated during 57 days in hospital on all his previous therapies until the tofacitinib was started. On day 3 after tofacitinib, he significantly improved. Of note, an IFX serum level before the colonoscopy done 8 weeks after beginning tofacitinib was undetectable: <1.7 μg/mL.

**TABLE 2.** Review of case series of pediatric patients with IBD treated with tofacitinib

Publication, number and age of patients	Patients	Duration of tofacitinib	Clinical remission	Colectomy	Response	Therapy at the end of follow-up
Dolinger et al (33) 2019	IBD-U1	5 wk	No	No	Primary nonresponse	UST
	IBD-U2	12.9 wk	Yes	No	Clinical remission off systemic corticosteroids	Combination TOFA/VDZ, rectal steroid enemas
	UC1	9 wk	Yes	No	Clinical response, no remission, slowly tapering steroids	Combination TOFA/VDZ
	UC2	11.7 wk	Yes	No	Steroid-free clinical remission	Combination TOFA/VDZ
	UC3	40.4 wk	Yes	No	Clinical remission off systemic steroids	TOFA oral budesonide
	UC4	10.3 wk	No	No	No remission achieved. Variable disease course	TOFA
	UC5	9 wk	Yes	No	Steroid-free clinical remission	TOFA
Twelve pediatric patients with biologic-refractory IBD	CD1	8.4 wk	No	No	Primary nonresponse	UST
						Diverting ileostomy
Median age 16 y (14–17 y)	CD2	8 wk	No	No	Primary nonresponse	
	CD3	58.8 wk	No	No	No remission achieved. Variable disease course	
	CD4	10.7 wk	Yes	No	Steroid-free clinical remission	Combination TOFA/VDZ
	CD5	5 wk	No	No	Primary nonresponse	Diverting ileostomy
Moore et al (35) 2021	UC (n = 14)	At week 6	n = 4/21	n = 4	Clinical response n = 10/21, 8 on steroids	
Twenty-one pediatric patients and younger adult with biologic-refractory IBD	IBD-U (n = 4)	At week 12 (17 patients completed induction)	n = 7/21		Clinical response n = 7/21, 5 on steroids	
Median age 18.4 y (15.1–19.1 y)	CD (n = 3)	At week 24 (15 patients on TOFA)	n = 7/17		Clinical response n = 12/17, 1 on steroids	
		At week 52 (10 patients on TOFA)	n = 7/17 with steroid-free clinical remission	n = 2	Clinical response n = 7/17, 0 on steroids	

CD = Crohn disease; IBD = inflammatory bowel disease, IBD-U = inflammatory bowel disease unclassified; TOFA = tofacitinib; UC = ulcerative colitis; UST = ustekinumab; VDZ = vedolizumab.

In addition, our patient was treated during the COVID-19 pandemic. He was exposed but not contaminated by the virus, although his parents developed a symptomatic COVID-19 infection during his admission. The safety documentation of tofacitinib during the COVID-19 pandemic is limited. Out of a total of 5000 patients with IBD and COVID infection, only 72 patients were treated with tofacitinib according to the Surveillance Epidemiology of Coronavirus Under Research Exclusion- database in contrast to 1581 patients treated with an anti-TNF. Two patients treated with tofacitinib had a fatal outcome with no significant difference with the other medications (38,39).

Interestingly, the effect of tofacitinib in recapturing response after treatment interruption was studied in patients who achieved clinical remission with tofacitinib in induction but were randomized to receive placebo in maintenance. The median time to treatment failure was 135 days after the interruption. A retreatment with 10 mg twice daily restored a clinical response in approximately three-quarters of patients within 2 months and the clinical response was sustained up to 1 year (40). Thus, due to its rapid onset, tofacitinib could be used for the induction of remission of severe UC, perhaps only as a short treatment and as a bridge to other maintenance treatments. In case of relapse, retreatment after discontinuation of therapy could be used safely and successfully. In the future, additional studies should make it possible to place tofacitinib in the recommendations for the management of IBD.

In conclusion, this case illustrates the potential successful use of tofacitinib in the treatment of a severe acute colitis in children. Without the use of tofacitinib, our patient would have undergone colectomy. After 6 months on tofacitinib, he remains in steroid-free clinical and histological remission. He has had no severe side effects. Prospective studies on the use, the efficacy and the safety of tofacitinib in pediatric UC are needed. In select cases, where therapeutic options are limited, tofacitinib may to be a good alternative to surgery due to its’ rapid efficacy and relative few adverse events, even in children. In the future, it may also be used as a bridging therapy to a more conventional biologic agent such as vedolizumab once endoscopic and histological remission have been obtained.

## ACKNOWLEDGMENTS

C.G. involved in conception of the study, acquisition and interpretation of data, drafting the article, and final approval of the version to be submitted. M.D. involved in interpretation of data, revising it critically for important intellectual content, and final approval of the version to be submitted. C.D. involved in conception of the study, interpretation of data, revising it critically for important intellectual content, and final approval of the version to be submitted.
